# Cross-species parallels in babbling: animals and algorithms

**DOI:** 10.1098/rstb.2020.0239

**Published:** 2021-10-25

**Authors:** Sita M. ter Haar, Ahana A. Fernandez, Maya Gratier, Mirjam Knörnschild, Claartje Levelt, Roger K. Moore, Michiel Vellema, Xiaoqin Wang, D. Kimbrough Oller

**Affiliations:** ^1^ Cognitive Neurobiology and Helmholtz Institute, Department of Psychology, Utrecht University, PO Box 80086, 3508 TB Utrecht, The Netherlands; ^2^ Museum für Naturkunde - Leibniz Institute for Evolution and Biodiversity Science, Invalidenstrasse 43, 10115 Berlin, Germany; ^3^ Laboratoire Ethologie, Cognition, Développement, Paris Nanterre University, Nanterre, France; ^4^ Animal Behavior Lab, Freie Universität Berlin, Berlin, Germany; ^5^ Smithsonian Tropical Research Institute, Balboa, Ancón, Panama; ^6^ Leiden University Centre for Linguistics, Leiden University, Leiden, The Netherlands; ^7^ Leiden Institute for Brain and Cognition, Leiden University, Leiden, The Netherlands; ^8^ Department Computer Science, University of Sheffield, Sheffield, UK; ^9^ Laboratory of Auditory Neurophysiology, Department of Biomedical Engineering, Johns Hopkins University, Baltimore, MD, USA; ^10^ School of Communication Sciences and Disorders, University of Memphis, Memphis, TN, USA; ^11^ Institute for Intelligent Systems, University of Memphis, Memphis, TN, USA; ^12^ Konrad Lorenz Institute for Evolution and Cognition Research, Klosterneuburg, Austria

**Keywords:** babbling, vocal learning, comparative vocal ontogeny, vocal play, vocal exploration, evolution of vocal communication

## Abstract

A key feature of vocal ontogeny in a variety of taxa with extensive vocal repertoires is a developmental pattern in which vocal exploration is followed by a period of category formation that results in a mature species-specific repertoire. Vocal development preceding the adult repertoire is often called ‘babbling’, a term used to describe aspects of vocal development in species of vocal-learning birds, some marine mammals, some New World monkeys, some bats and humans. The paper summarizes the results of research on babbling in examples from five taxa and proposes a unifying definition facilitating their comparison. There are notable similarities across these species in the developmental pattern of vocalizations, suggesting that vocal production learning might require babbling. However, the current state of the literature is insufficient to confirm this suggestion. We suggest directions for future research to elucidate this issue, emphasizing the importance of (i) expanding the descriptive data and seeking species with complex mature repertoires where babbling may not occur or may occur only to a minimal extent; (ii) (quasi-)experimental research to tease apart possible mechanisms of acquisition and/or self-organizing development; and (iii) computational modelling as a methodology to test hypotheses about the origins and functions of babbling.

This article is part of the theme issue ‘Vocal learning in animals and humans’.

## 1. Introduction

Vocal production learning (VPL) is the ability to modify the structure of vocalizations as a result of hearing those of others [1]. The motor learning phase often starts with a plastic stage when highly variable vocalizations are produced early in development. In humans this stage is commonly referred to as babbling, a term sometimes also applied to non-humans. The timing and substages of babbling and the role of learning versus predisposed mechanisms appear to differ across species; however, common patterns have been observed as well. Based on these observations, we propose the following cross-species definition: babbling is an exploratory stage in vocal development marked by many variable and repetitive vocalizations, for which production does not require a specific social or functional context, suggesting exploration.

The sounds of babbling are produced in large amounts by young animals in isolation, but unlike ‘isolation calls’ do not have a specific communicative function. In addition, babbling behaviour does sometimes occur in social interaction. Yet even babbling that is not socially directed may produce reactions from listeners (in, for example, human babbling or zebra finch (*Taeniopygia guttata*) subsong) [2–4]. Babbling is a precursor to the adult form of vocal communication in the sense that the sounds produced in babbling incorporate acoustic features required in the adult vocal system. In the most advanced babbling forms across various taxa, syllable-like elements emerge that often constitute well-formed exemplars of syllable-like elements of the adult system. In the human case, those well-formed syllables are called ‘canonical syllables’.

Juvenile production of sounds not found in the adult repertoire during begging, distress or greeting, as for instance occurs in rats [5], naked mole rats [6] and grey mouse lemurs [7], is not considered here. Such sounds superficially resemble babbling but differ from it in structural composition (including only variable juvenile syllables rather than juvenile and adult syllables) and in that they show context specificity, while babbling does not. Variation in vocalizations during development due to purely physical or physiological change is also not considered babbling here. While such changes may also be relevant for vocal ontogeny and may interact with vocal learning, they are not the focus of this review.

It has been proposed that the functions of babbling include a form of practice or exploration, facilitating vocal imitation learning [3]. In some species, there is evidence that babbling is self-rewarding, a kind of ‘vocal play’ [8,9]. A similar notion has been proposed in computational modelling of vocal development, with the idea of curiosity-driven learning [10].

Fitch [11] hypothesized that babbling may be a prerequisite for complex vocal learning. Here, we discuss evidence and counter-evidence for this hypothesis. The idea requires that (i) all vocal learners have babbling in infancy and further implies that (ii) babbling may be absent in non-learners. Although much research remains to be done, a preliminary review suggests at least support for the first claim. Counter-evidence for the hypothesis would be the existence of vocal learners without a babbling phase. Literature for counter-evidence is lacking or inconclusive, but there are candidate species that require further investigation before we are able to reject or accept the hypothesis convincingly.

One purpose of the present paper is to compare and contrast species with regard to babbling, both its stages and its hypothesized functions, over the course of its development and in relation to the emergence of mature vocal production. The strength of comparative research lies in identifying similarities and differences, in order to pinpoint possible common mechanisms and possible changes in evolutionary history. To this end, we describe vocal developmental stages for several babbling animal groups: humans, avian vocal learners, several non-primate mammals including bats and non-human primates (marmosets). Vocal-learning mechanisms differ between species and range from imitation learning and incorporation of new sounds in many songbirds and bats, to acoustic change of existing calls, presumably through parental auditory feedback in marmosets. We also discuss a few (possibly) non-vocal-learning species in which babbling might occur. However, an exhaustive comparison of all vocal developmental phenomena is beyond the scope of this review. In addition, we contrast learned and non-learned vocalizations within and between species in order to address the role of vocal learning by auditory input versus self-organization and exploratory vocalization. Finally, we review computational modelling directed at testing potential mechanisms of vocal exploration and learning.

## 2. Babbling in humans

### (a) Background

Human infants produce massive numbers of ‘protophones’, the presumed precursors to speech [12], across the first year. The precursor status of protophones has been documented by the observation of features of speech being systematically incorporated into the protophones by infants, in stages across the first year [12,13]. Protophones constitute more than 4/5 of all infant utterances [14]. They include identifiable phonatory types, among them squeals, growls, vowel-like sounds, as well as canonical babbling [9,13], all used with varying functions; each protophone type can be expressed with positive, negative or neutral facial affect on different occasions [15,16]. Neutral affect accompanies the great majority of protophones, a pattern suggesting vocal exploration. All-day recordings sampled randomly across the first year and coded by human listeners suggest infants produce approximately 3500 protophones per day [14], in both face-to-face interaction and even more frequently when infants are directing the sounds to no-one [17], again suggesting exploration.

### (b) Vocal stages

Vocal stages over the first year have been described as including the following five, where protophones: (i) are differentiated primarily by phonatory characteristics; (ii) include primitive supraglottal articulatory actions, where the divergence from the at-rest vocal tract state is minimal; (iii) are differentiated by both extensive openings and closings of the supraglottal tract during phonation, and where playful repetition of utterance types becomes apparent; (iv) come to include well-formed ‘canonical’ syllables often produced in sequences such as ‘baba’ or ‘nana’; and (v) begin to be adapted as early words [12].

**Figure 1.**
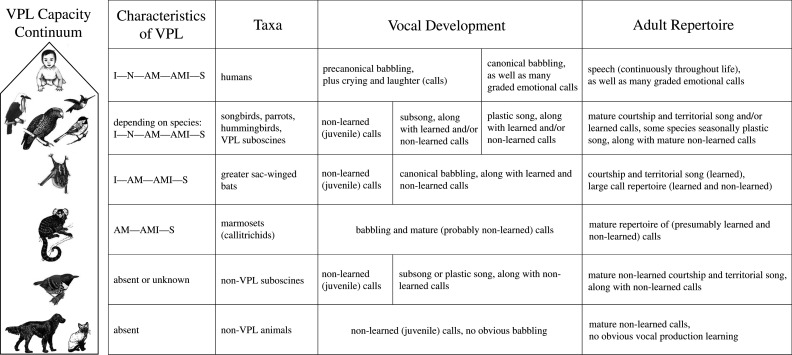
A hypothesized comparative summary of relations between babbling and vocal production learning (VPL) in selected taxa. The top four rows represent taxa for which at least some form of vocal learning is known. The first column represents known vocal-learning characteristics. I, imitation; N, incorporation of novel sounds; AM, acoustic modification; AMI, auditory–motor integration; S, social feedback. Columns represent crude developmental stages (width is arbitrary). All vocal learners show a stage of babbling as well as calls, but babbling onset may differ. Although vast numbers of vertebrates produce vocalizations communicatively, many have never been reported to show VPL or babbling. The term ‘calls’ in the figure refers to vocalizations that are communicative already at developmental onset (in contrast to song), with varying amounts of VPL. Calls may or may not be part of babbling depending on the call and the species. Song in songbirds, hummingbirds and greater sac-winged bats is shaped by VPL. The figure summarizes data from a variety of species that appear to support the working hypothesis that babbling or some form of precursor vocalization in infancy may be a requirement for VPL, which often results in complex mature vocal repertoires.

These stages can be simplified to a first stage of precanonical protophones, a second including canonical ones as well and a final stage in which canonical syllables become adapted to be used as words (figure 1). The number of syllable types that can form parts of words is usually small (approx. 3–6) through the first year [18], expanding thereafter [19]. Even after words enter the infant repertoire, protophone production continues through to at least the age of 16 months.

### (c) Vocal features, exploratory

The exploratory pattern of infant protophones is reminiscent of other infant activities, for example, play with objects that are repetitively manipulated and examined [20,21]. Vocal play also similarly involves repetitive production of similar vocal types. Given this similarity with object play, self-motivated protophone production has also been called ‘vocal play’ [22] and has been thought of as a kind of practice, just as object play has been treated as a learning endeavour.

### (d) Vocal features, social

Protophones in the first year are frequently produced with no apparent social intention and frequently even when infants are alone. Yet the sounds can also sometimes be used with unambiguous social directivity, in some cases to call for attention and in others to express complaint or explicitly to solicit help. Perhaps most importantly, they can be used in face-to-face interaction [23–25], where sharing of affect appears to be a primary function. Such vocal interaction with no sign of distress, often with sustained eye contact, has not to our knowledge been reported in any other species. Vocal interaction in infancy is a foundation for and appears to facilitate learning of the ambient language by shaping language input from caregivers [26]; infants from five months expect a response from caregivers to their socially directed vocalizations [27], and caregivers simplify speech in contingent responses to both canonical and precanonical babbling, using fewer unique words and shorter utterances [26,28].

### (e) Babbling in deaf infants

Canonical babbling is severely delayed in deaf infants, but surprisingly, all the precanonical sound types found in hearing infants in the first six months are also found in the small number of deaf infants that have been evaluated longitudinally [29]. Equally surprising is that the amount of protophone production appears to be no lower in deaf than in hearing infants across the first year [30,31], another fact hinting at the endogenous motivation for protophone production.

### (f) Babbling after infant tracheostomy

Extensive exercise in babbling during the first year does not appear to be a requirement for learning to talk, however. There are infants with laryngeal anomalies who cannot vocalize because they require tracheostomy to breathe. After surgical repair, often around the end of the second year, allowing the infants to breathe through their supraglottal tracts, they often go on to learning to talk within a few months as long as there are no secondary handicaps [32–34]. How much babbling is required, if any, remains uncertain. Yet even if extensive babbling is not an absolute requirement of learning to talk, we cannot rule out the possibility that it supports acquisition.

### (g) Robustness of human babbling and possible variations across cultures

Human infant babbling has been observed across a wide variety of cultures and ambient languages and shows substantial robustness in onset of stages [12]. Even with premature birth and language handicapping conditions, precanonical babbling appears to be relatively normal, and while the onset of canonical babbling is sometimes delayed, it is not prevented. This babbling robustness applies to conditions as diverse as Down syndrome, Williams syndrome, autism and deafness [12]. Low socio-economic status correlates with low volubility in babbling and may delay canonical babbling onset, but does not prevent either precanonical or canonical babbling [35]. Little is known as yet about volubility or stage onset for babbling in foraging or minimally agricultural communities.

### (h) Roots of human babbling

Babbling is thought to have evolved as an endogenous activity under the influence of hominin altriciality, large group sizes and cooperative breeding [36–38]. In particular, altriciality has been hypothesized to have produced selection pressure on vocal fitness signalling, yielding infants more inclined to produce exploratory and interactive vocalizations, signalling their wellness and social motivation to caregivers. Babbling is thought to have formed a foundation for the subsequent evolution of more elaborate capabilities required in vocal language [39].

## 3. Babbling in avian species

### (a) Background

Songbirds have long been studied for their vocal-learning capacities and parallels with human vocal ontogeny. Parrots and hummingbirds are also known vocal learners, but have been much less studied. We describe commonly studied examples of vocal-learning birds and compare them with suboscines, a clade related to oscines, where both vocal learning and non-vocal-learning species exist.

### (b) Vocal stages: babbling and early song development

Birdsong motor development starts with an early highly variable stage, termed subsong, followed in some species by rhythmic sequences of basic vocal units, reminiscent of human precanonical babbling, with relatively amorphous acoustic structure (figure 1) [3]. As in the precanonical babbling of deaf human babies, subsong initiation also occurs in deaf birds, suggesting it is independent of auditory input. However, the song remains flexible and maturation into fixed syllables and sequences does not occur in deafened songbirds, since auditory–vocal exploration is prevented [40]. Subsequently, an input-dependent increase in acoustic structure occurs in most juvenile songbirds and eventually leads to distinct acoustic categories, first with variable sequence structure in plastic song, reminiscent of human syllables in canonical babbling, and subsequently developing into learned adult crystallized song (figure 1) [3]. A very similar pattern of vocal development has been described for hummingbirds and parrots, two groups of birds where many species show VPL [41–43]. Some species temporarily increase their song repertoires during development, with subsequent selection and attrition of song notes (e.g. chaffinch (*Fringilla coelebs* [44]) and white-crowned sparrow (*Zonotrichia leucophrys* [45])).

Although external auditory input usually shapes the final songs, it is not necessary to initiate subsong. Deafened birds or birds raised in complete social isolation will initiate song development [46,47], suggesting that the motivation to start singing is internally guided. Concordantly, large quantities of subsong without social context are observed in songbirds as well as parrots [41,43]. In addition, subsong is often considered exploratory [48], a form of self-rewarding vocal play [8].

In addition to the learned song, several songbird species also produce learned and/or non-learned calls, which are vocalizations with specifiable functions from their first use, whereas song is only used for mating and territory defence as the birds approach adulthood. Song notes as well as adult calls can emerge from earlier hatchling or fledgling calls serving as precursors [49], in the same way that one (proto)syllable can diverge into two new ones [50]. If early calls are precursors of song syllables, variable and produced without specific social context, they can potentially be considered as part of subsong or babbling-like behaviour (e.g. food begging calls in the chipping sparrow (*Spizella passerina*) [51]). However, this likely differs between calls and species and further systematic studies are necessary.

### (c) Vocal stages: babbling in adulthood

Babbling-like utterances have been described for many avian species; we are not aware of any songbird species that does not show some form of babbling-like precursor to the song. In fact, even some songbirds that are not generally known to sing, such as female canaries (*Serinus canaria*), have been shown to occasionally produce vocalizations that are structurally similar to subsong throughout life [52]. In addition, fully adult female canaries can still be stimulated to develop full songs, going through the archetypical phases of VPL, including subsong and plastic song, if treated with the hormone testosterone [53], indicating that babbling-like vocal exploration is not necessarily limited to early development.

Some seasonally breeding songbirds, such as canaries [54] and starlings (Sturnidae) [55], have been reported to incorporate new elements into their songs throughout adult life. Although the incorporation of novel sounds into existing song repertoires seems always to be preceded by some vocal practice, such alterations to the adult song do not lead to a return to a subsong phase [53,54]. Subsong appears primarily to initiate the first time of VPL, be it during juvenile development in early life, or in some species (e.g. chaffinch) in adolescence at the start of the first breeding season, or in adulthood with hormone treatment. Thus, subsong may not be a requirement for modifications to acoustic structures in all cases, but instead may function specifically to probe vocal capabilities in preparation for vocal learning.

### (d) Babbling in birds closely related to the oscines

The clade of suboscines, the closest relatives of oscines, consists of both vocal learners and non-learners. Data on vocal development in this group are limited but highly relevant. Suboscine vocal learners such as bellbirds (*Procnias*, [56]) produce aberrant song if raised in isolation, whereas vocal non-learners such as eastern phoebes (*Sayornis phoebe*) sing the normal song even when deafened before song-learning onset and [57] and spotted antbirds (*Hylophylax naevioides*) show little or no evidence of abnormal song when reared in isolation or with heterospecific tutoring [58]. Regardless, at least eastern phoebes and spotted antbirds do show a long period of the plastic song during development, produced in high amounts and with high variability, possibly similar to the plastic song in songbirds [58]. Touchton *et al*. [58] describe early song stages as ‘babbling’ and ‘subsong’ but based on limited information the developmental pattern seems more comparable to the plastic song, since ‘prototypes’ (rudimentary songs or calls) seem to be present from the onset of singing. However, at this point, we can only speculate, and more research is necessary on the acoustics and the social context to draw any conclusions in this respect.

## 4. Babbling in non-human mammals

### (a) Background

Here, we focus on the greater sac-winged bat and the giant otter, which exhibit a vocal practice behaviour that shares certain aspects with human infant babbling. Several other non-human mammals, such as bottlenose dolphins, belugas and two other bat species, are also promising candidates.

### (b) Babbling in bats

Greater sac-winged bats (*Saccopteryx bilineata*) are capable of VPL [59] and have a large vocal repertoire, including male song. Directly after birth, pups only produce isolation calls. Around two weeks of age, pups also produce precursors of adult vocalizations in so-called babbling bouts [60,61]. Pup babbling constitutes multisyllabic vocal sequences composed of adult-like vocalizations (precursors of distinct adult syllable types) mixed with pup-specific vocalizations such as isolation calls (figure 1). During babbling, pups acquire a part of the adult vocal repertoire through VPL, namely the syllables of the territorial song [60,61]. Pup precursors of territorial song syllables gradually converge towards the territorial song of tutor males, irrespective of relatedness and pup sex. Isolation calls are also influenced by VPL as they converge towards isolation calls of fellow pups, resulting in a learned vocal group signature [59]. Whether the remaining syllables of the adult vocal repertoire are acquired through VPL remains unknown. Pup babbling is not associated with a specific behavioural context [60] and occurs until the age of 10 weeks, at which point weaning occurs, and babbling stops abruptly. Subadult bats produce vocalizations of the adult vocal repertoire only. Likely, non-mutually exclusive functions of babbling in *S. bilineata* (i) are vocal practice to refine control over vocal motor actions, especially for syllable types acquired through vocal imitation, and (ii) support eliciting maternal care (e.g. nursing).

### (c) Babbling in otters

Giant otters (*Pteronura brasiliensis*) are cooperative breeders and possess a large vocal repertoire, but it remains unclear if they are capable of VPL [62]. They are reproductive at an age of 2–3 years and either stay in their natal group as non-breeding helpers or disperse. Cubs are vocally active from birth on. Inside the den, they engage in a conspicuous vocal practice behaviour [62]. Cubs produce a subset of precursors to adult vocalizations from birth on (presumably exploring vocal features) as well as vocalizations exclusively produced by cubs. The entire adult vocal repertoire consists of at least 15 distinct vocalization types and is further enlarged by gradations between them. It is present at three months of age, but acoustic parameters continue changing until 6–12 months [62]. In giant otters, babbling probably constitutes motor practice (even though it is currently unknown if VPL occurs) and may also have the advantage of eliciting care from group members [62].

### (d) Additional potentially babbling mammals

Several other mammals, for instance, the common bottlenose dolphin (*Tursiops truncatus*), the beluga whale (*Delphinapterus leucas*), the Egyptian fruit bat (*Rousettus aegyptiacus*) and Horsfield's leaf-nosed bat (*Hipposideros larvatus*), exhibit vocal behaviours that could potentially be classified as babbling if described in more detail (e.g. if comparisons with the adult vocal repertoire were available along with detailed information on social context). During ontogeny, infant bottlenose dolphins and beluga calves both engage in highly variable vocal sequences, including exclusive infant calls and adult-like variants [63,64]. Egyptian fruit bat pups produce highly variable immature social calls during ontogeny, thereby transitioning from isolation calls to mature adult calls [65]. Horsfield's leaf-nosed bat pups produce a mixture of juvenile and adult-like syllables which gradually merge into adult syllable types [66]. In contrast to the three species mentioned previously in this paragraph, it is currently unclear if Horsfield's leaf-nosed bat is capable of VPL.

## 5. Babbling in New World primates

### (a) Background

Babbling in infants and juveniles has been documented in two species of New World primates: pygmy marmosets (*Cebuella pygmaea*) and common marmosets (*Callithrix jacchus*), both of which are highly vocal [67]. Evidence of babbling in Old World primates has been lacking. The most extensive and systematic analysis of babbling behaviours in monkeys was performed with captive pygmy marmosets [68]. Babbling in captive common marmosets has been reported by Pistorio *et al*. [69] and in a study of the effects of auditory deprivation on vocal development [70]. Gultekin & Hage [71] also have reported babbling in common marmosets in a more recent study, evaluating parental interaction during vocal development.

### (b) Similarities and differences between the two marmoset species

Overall, the properties of babbling behaviours are remarkably similar in pygmy marmosets and common marmosets. Babbling in both species is characterized by sequences of repetitive, rhythmical vocalizations. The call types contained in a babbling sequence usually consist of a subset of call types used by adults of the species or of variations on the adult types. However, there are plenty of instances of ‘immature calls’, where individual vocalizations in a babbling sequence significantly deviate from those of adult call types (fig. 6C in [69]). In addition, nearly all animals of both species start babbling soon after birth (as early as the first week) and continue into the juvenile stage. Snowdon & Elowson [72] reported that babbling bouts of pygmy marmosets continued to appear through the age of puberty, but at a reduced rate from that seen in infancy, with decreasing proportions of immature calls and variations on adult calls (figure 1). Babbling was rarely observed in adult pygmy marmosets [72].

A general trend in vocal development of common marmosets is that the frequency of all call productions, including babbling, decreases steadily with age, from 400 to 500 calls per hour at four weeks to approximately 200 calls per hour at 15 weeks, and to below approximately 50 calls per hour at 30 weeks [70]. Up to six to seven weeks, baby cry vocalizations and babbling are the most common forms of vocalizations uttered by common marmosets. However, the babbling abruptly disappears, usually by the seventh week, and the use of cry calls fades gradually and disappears entirely by 10–11 weeks. Interestingly, in a study of common marmosets that lost hearing at an early age, babbling was observed long after the animals were fully grown adults [70]. It is also interesting that although baby cry vocalizations decline at a similar rate in both deaf and hearing siblings, some deaf marmosets continue to cry even at the age of 1 year. This result appears to be at variance with a prior report [69], which had indicated crying did not occur in hearing marmosets in isolation after the age of 25 weeks.

### (c) Functions of babbling in marmosets

What could the functions of babbling in marmosets be? In both infant and juvenile marmosets, babbling may provide vocal practice as well as attract attention from parents and other group members [72]. Elowson *et al*. [68] pointed out similarities between babbling in human infants and pygmy marmosets. Both species produce well-formed, recognizable phonetic or syllable-like units similar to adult-like calls or syllables. Like human infants, marmoset infants babble without an obvious communicative function other than vocal exploration or play. While most vocalizations in marmoset babbling appear similar in acoustic structure to calls produced by adult marmosets, babbling marmosets do not display behaviours corresponding to social functions often associated with adult vocalizations. There are, of course, important differences between human speech and marmoset vocalizations. For example, marmoset calls do not appear to be like words, which can be formed in human language by recombination of smaller syllabic or phonemic units, nor is there any evidence that marmosets form complex sentences from calls. Because there has not been a direct quantitative comparison of babbling in humans and marmosets, we do not know how similar or different their patterns of babbling are. It is also not yet certain whether immature calls in marmoset babbling can be treated as analogous to the precanonical babbling of human infants. It remains possible that the driving forces of babbling in both humans and marmosets are similar: (i) vocal practice preparatory for the adult repertoire and (ii) attracting attention from potential caregivers. Notably, there are many potential caregivers for infants of both humans and marmosets, because both are cooperative breeders [36].

## 6. Computational approaches to vocal learning

### (a) Background

It has long been accepted that a productive approach to understanding how an observed system might function internally is to construct a mechanism that exhibits the same behaviours (‘What I cannot create, I do not understand’, Richard Feynman). In modern times, this usually involves the creation of appropriate computational models, that is, algorithms that attempt to replicate the processes of interest and thereby provide a functional testbed for selecting among alternative hypotheses. In the case of systems that ‘learn’, recent times have seen huge developments in the fields of artificial intelligence and machine learning, primarily arising from advances in multi-layered artificial neural networks, an approach known as ‘deep learning’ [73]. It is, therefore, no surprise that a few researchers have started to apply these techniques to problems in bioacoustics [74], particularly for automatic call detection and classification [75,76]. However, as yet, there are few studies that apply such algorithms to vocal learning itself, and of those, all have been concerned with modelling the acquisition of vocal abilities by humans, and none, to our knowledge, has addressed vocal learning in other animals or in a general cross-species approach.

### (b) Computational learning models

Perhaps the earliest computational model of vocal learning in human infants is DIVA [77,78], a neural-network model that uses babbling to simulate learning of phonetic-to-orosensory and orosensory-to-articulatory mappings. Contemporaneous work by Bailly [79] showed that a computational model of the articulatory system could learn to speak in four developmental steps: babbling, imitation, phonemic shaping and rhythmic coordination. The key principles underlying such models are exploration and imitation [80,81]. However, the imitation phase in human beings is problematic owing to the large physical difference between the infant-learner and the adult-teacher vocalizations. Howard & Messum [82] addressed this ‘correspondence problem’ by implementing a computational simulation of infant speech development based on reformulated feedback from the caregiver, i.e. implementing a form of ‘reinforcement learning’, and a similar approach was adopted by Warlaumont [83] and Rasilo & Räsänen [84].

### (c) Motivated learning

Of particular importance in constructing computational models of learning is the ‘objective function’ that is being optimized during exploration, i.e. how does the model judge the quality of its behaviours? Typically, this will involve some form of ‘closed-loop’ feedback that provides the information needed to adapt motor control strategies in an appropriate direction. In modelling human infant vocal learning, some researchers have found success in casting this as an intrinsic motivational drive to maximize progress in competence, referred to as ‘curiosity-driven learning’ [85,86].

### (d) Developmental approaches

Finally, although there are only a few extant computational models of vocal learning, there is considerable interest in the general principles of motor learning in the field of ‘developmental robotics' [87]. Of particular relevance are approaches that invoke a babbling phase of self-exploration in order to learn an ‘inverse model’ of the relationship between motor controls and subsequent behaviour [87–91]. In particular, there are important relationships between (i) the number of ‘degrees-of-freedom’ (DoF) of a system's morphology, (ii) the state-space of possible behaviours to be explored and (iii) the learning policy required to calibrate the control mechanisms to a target level of accuracy (judged intrinsically or extrinsically). The fact that motor babbling offers significant benefits in robotics provides evidence that such computational principles are somewhat independent of the learning agent (whether living or non-living), and thus could be applied to the investigation of animal vocal development.

### (e) Open questions

Therefore, what appears to be needed in the field of vocal learning is a clear enumeration of the research questions (box 1) that could be addressed using insights from existing computational models of learning in general, and motor learning in particular.

Box 1. **Relevant questions from a computational modelling perspective:**

1. What is the advantage of repetitive babbling relative to a purely random exploration strategy for calibrating a control mechanism?

2. How might the emergent spectro-temporal structure of babbling be conditioned on the under-actuated elastic agonist–antagonist morphology that is characteristic of living systems?

3. What is the optimum progression of vocal patterning given different strategies for sampling the different degrees-of-freedom of the motor apparatus?

4. What motor control parameters are adapted and what objective function is being optimized during learning?

5. What is the nature of the feedback that permits such optimization?

6. What are the consequences of a morphology that changes over time developmentally for recalibrating control?

## 7. Discussion

### (a) Similarities and differences in babbling across species

The comparisons made here suggest that all babbling species considered may follow a similar developmental trajectory. Although important details on the developmental trajectory are missing for many species, vocal development appears to fall into two or three phases (figure 1) for those species that have been studied most intensively. The first is an exploratory phase in which variable sounds are produced, independent of a specific social context. In some species (e.g. songbirds, bats), the onset of this stage is not immediately after birth but follows a period characterized by mostly juvenile-specific innate calls. Some species may produce only innate calls during infancy, whereas others produce mostly learned calls (e.g. parrots) or a mix of both (e.g. zebra finch). In avian vocal learners and humans, variable and immature sounds continue to develop, yet become more structured to form relatively well-defined acoustic units. The final stage is composed of adult vocalizations such as songs or speech, accompanied by adult calls.

One of the differences among species concerns the timing of these stages, even if relative developmental time is considered. For example, in humans and bats, precursors to canonical babbling appear in early infancy, whereas subsong in songbirds and hummingbirds usually only appears around fledging from the nest or later, and in some species even in adolescence (just before the breeding season, figure 1). In addition, exploratory vocal development can extend into maturity, as in humans (who can practise vocalization throughout life), whereas in other species babbling can end abruptly, at weaning for example in bats and marmosets.

Although the great majority of documented babbling species show VPL, vocal imitation does not necessarily play a major role during this developmental stage in all species. For instance, the grasshopper sparrow, a songbird, requires auditory input by tutoring, but does not accurately imitate the specific tutor [92]. Furthermore, while the capability to imitate is a logical necessity for language learning, vocal imitation events account for only a miniscule proportion of babbling in human infants. Similarly, subsong in songbirds is also experience-independent initially and, as in human infants, occurs even in cases of deafness.

### (b) Babbling with limited vocal learning

Species in which there is limited or no VPL sometimes still show variable vocalizations during development, without specific social context. In non-vocal-learning suboscines, a phase comparable to the plastic song of songbirds sometimes appears to be present. Some basic calls or rudimentary song occur very early in some species without VPL (e.g. in spotted antbirds and eastern phoebes) but are not yet structured to include the adult form and sequence. Importantly, at least in eastern phoebes, vocal plasticity and maturation are independent of hearing. Thus, early plasticity in these non-vocal learners is more likely to be guided by physiological processes as suggested for the non-VPL quail, but during a longer developmental period in eastern phoebes than quail [93,94]. The lack of necessity for auditory feedback complicates the question of babbling as a form of exploratory behaviour. Auditory–vocal exploration would seem to require auditory–vocal feedback, but there are also kinaesthetic consequences of vocalization that may be the focus of exploration. Data on development and the extent of vocal learning are limited for many suboscine species, including the spotted antbird, so a firm conclusion cannot be drawn at this time about the role of sensory feedback in babbling and VPL. Future research should reveal for many species whether developmental vocal behaviour has an exploratory nature that can be classified as babbling.

Similar to non-VPL birds, marmosets also produce adult-like calls within babbling sequences, perhaps shaping them for adult usage. Auditory input affects developmental patterns and vocal interaction in marmoset infants, but not apparently the acoustic structure of final call production [71]. Future work on marmosets and non-VPL birds in comparison with imitating species could reveal potential differences in babbling patterns.

### (c) Babbling as independent of VPL or as a possible prerequisite

The hypothesis that babbling is a prerequisite for vocal learning [11] is supported by the lack of reports on species that show VPL without (at least rudimentary) babbling. However, there are approximately 4000 species of songbirds alone, with developmental research on only a very small proportion. It is uncertain to what extent babbling is necessary at the high frequency of occurrence often observed during development. A study on zebra finches prevented from vocal production by a weight on their neck during development showed they were still able to learn the song, even in adulthood. However, even though song learning was postponed, it did start with subsong or plastic song, indicating that some vocal exploration and/or practice may be necessary [95]. Similarly, human infants unable to babble owing to laryngeal anomalies that require tracheostomy are reported to be able to produce words sometimes within two or three months after surgery; while there appears to be an intervening brief period of babble-like practice, research has not produced an unambiguous conclusion that babbling is required [32–34]. Moreover, studies in adult parrots [96] and adult canaries [53,54] rapidly learning new syllables or calls after their first season suggest only a limited plastic stage is necessary once the vocal system is ‘calibrated’. This suggests that under certain conditions, learning can take place without an extensive duration of babbling. However, in these cases, exploration and/or practice of the vocal organ has already occurred during development. Therefore, we cannot yet draw a firm conclusion about the extent to which babbling is required for vocal learning. However, some exploration of the vocal organ and practice appears to be necessary for animals to produce novel sounds. Additional factors such as physical maturation and hormones could explain the protracted period, which also occurs in non-learning eastern phoebes [94]. Furthermore, while *the initiation* of babbling does not seem to be dependent on auditory input, progression to more mature vocalizations and distinct categories does seem to be dependent on auditory–motor feedback.

It should be noted though, that data on vocal development and vocal learning in suboscines are very limited, and much more research is necessary to verify the relation between babbling and vocal learning in many species. Many mammalian potential babblers require more systematic investigation, with detailed acoustic analysis of juveniles compared with adults, and specific attention to context specificity and exploratory behaviour. The same holds for research on parrots and suboscines, since babbling and vocal-learning data are available for only very few species. Hummingbirds also deserve much more study, since it appears in some cases that the song itself has evolved, disappeared and then evolved again, yet research on vocal development in hummingbirds is extremely limited [97]. In two exceptional songbird species, the development of complex vocal repertoires has been reported in the absence of external auditory input: sedge warblers (*Acrocephalus schoenobaenus*) [98] and grey catbirds (*Dumetella carolinensis*) [99]. Unfortunately, vocal ontogeny has not been described for these birds as far as we know, but may shed light on the relation between vocal learning and babbling.

Whether or not babbling appeared before or after VPL in evolution may be informed by patterns of appearance, loss and renewed appearance of VPL. Multiple occasions of loss and reappearance of singing across evolution have been reported in hummingbirds [97]. This provides an excellent opportunity to study the evolutionary order of babbling and vocal learning. The finding that calls are often present in babbling along with the fact that some species show vocal learning of calls may provide support for the idea that vocal learning begins in evolution with call learning, which may itself be an aspect of babbling. More research is clearly needed on evolutionary history in various species, with and without babbling, and with and without VPL, in order to assess the possibility that babbling is required for VPL.

### (d) Potential functions of babbling

Among the proposed functions of babbling in the species considered here are vocal exploration and/or practice. This behaviour may be described as the (computationally inspired) notion of ‘calibration’. Vocalizations during early development are often highly variable, produced at high rates and most importantly, often without specific (social) context and are even produced in isolation. These facts support the idea of an internal reward system for vocal exploration [8]. Still, there may be a role for practice, even limited practice, in babbling. In species where the basic building blocks for the adult repertoire are already present from the start, such as in suboscines, marmosets and songbirds after their first seasons' plastic stages, an extensive exploration of and practice with the vocal apparatus (i.e. ‘calibration’) does not seem necessary. Yet the (limited) vocal variation that does occur may well tune the system, optimizing and sequencing vocalizations towards adult target vocalizations. Thus, there is currently no strict line to draw between exploration and practice in babbling, and tying down a mechanistic difference remains to be determined in future research (with insights from computational models and machine learning).

In addition, in humans, marmosets and bats, babbling may well have social functions. Babbling appears to be self-rewarding, and some have suggested that it may elicit higher levels of care giving, thus supplying a selection mechanism for babbling independent of a possible practice function. In the sac-winged bat, isolation call syllables, used to solicit maternal care, are integrated into babbling sequences [60,61]. It has been argued that human infants signal well-being by babbling both in face-to-face interaction and when potential caregivers are out of sight [17]. Also in marmosets, babbling has been interpreted as being used to attract attention from parents and alloparents [71]. In these cases, the same kinds of vocalizations are also produced in the absence of any social context, a crucial criterion for babbling. In songbirds, a social function such as attention seeking has not explicitly been reported, although direct consideration of the possibility of social signalling may deserve attention in future songbird research. However, there appears to be no doubt there is a role for social interaction in the form of social feedback from parents, feedback that contributes to (but is not an absolute requirement for) shaping vocalization in both songbirds and humans [2].

### (e) Future directions for research, including computational tests of babbling and VPL

Research on babbling and VPL has been extensive, and yet there are still numerous open questions. Among them are the questions listed in box 2. Only a small number of species have actually been studied at close range with longitudinal observational methods as well as experimentation to determine the nature and extent of babbling or babbling-like behaviour. The time is ripe for converging studies using increasingly sophisticated technologies for observation and experimentation along with empirical existence-proof tests through computational modelling. Research that triangulates insights from computational modelling and machine learning (box 1) with observational and experimental studies across species promises to offer major new opportunities to investigate babbling, its functions and its possible role in VPL.

Box 2. **Open questions for future directions:**

1. Is imitation necessary for novel vocal category formation?

2. Is it necessary to practise in order to refine vocal control?

3. Is VPL an evolutionary consequence of babbling?

4. Can we distinguish self-organizational category development from input-based learning?

5. Is it possible to model the relation between the complexity of babbling and the complexity of adult vocal repertoires?

## Data accessibility

This article has no data.
